# Developmental and Pathological Changes in the Human Cardiac Muscle Mitochondrial DNA Organization, Replication and Copy Number

**DOI:** 10.1371/journal.pone.0010426

**Published:** 2010-05-03

**Authors:** Jaakko L. O. Pohjoismäki, Steffi Goffart, Robert W. Taylor, Douglas M. Turnbull, Anu Suomalainen, Howard T. Jacobs, Pekka J. Karhunen

**Affiliations:** 1 Department of Forensic Medicine, Medical School, University of Tampere, Tampere, Finland; 2 Institute of Medical Technology, University of Tampere, Tampere, Finland; 3 Mitochondrial Research Group, Institute for Ageing and Health, The Medical School, Newcastle University, Newcastle upon Tyne, United Kingdom; 4 Research Program of Molecular Neurology, University of Helsinki, Helsinki, Finland; Hospital Vall d'Hebron, Spain

## Abstract

Adult human heart mitochondrial DNA (mtDNA) has recently been shown to have a complex organization with abundant dimeric molecules, branched structures and four-way junctions. In order to understand the physiological significance of the heart-specific mtDNA maintenance mode and to find conditions that modify human heart mtDNA structure and replication, we analyzed healthy human heart of various ages as well as several different heart diseases, including ischemic heart disease, dilated as well as hypertrophic cardiomyopathies, and several mitochondrial disorders. By using one- and two-dimensional agarose gel electrophoresis, various enzymatic treatments and quantitative PCR we found that in human newborns heart mtDNA has a simple organization, lacking junctional forms and dimers. The adult-type branched forms are acquired in the early childhood, correlating with an increase in mtDNA copy number. Mitochondrial disorders involving either mutations in the mtDNA polymerase γ (PolGα) or mtDNA helicase Twinkle, while having no obvious cardiac manifestation, show distinct mtDNA maintenance phenotypes, which are not seen in various types of diseased heart or in mitochondrial disorders caused by point mutations or large-scale deletions of mtDNA. The findings suggest a link between cardiac muscle development, mtDNA copy number, replication mode and topological organization. Additionally, we show that Twinkle might have a direct role in the maintenance of four-way junctions in human heart mtDNA.

## Introduction

The heart is the most energy demanding tissue in the human body consuming 0.1 ml O_2_/g per minute – an amount that is only surpassed by insect flight muscles [1]. Heart has also an impressive reserve capacity; human cardiac muscle oxygen consumption can rise four-fold without any effect on the steady-state ATP levels. There is also no accumulation of oxygen debt, as seen in skeletal muscles under prolonged exercise. The continuous circulatory pumping of blood by the heart as well as the maintenance of ion homeostasis in cardiomyocytes is dependent on healthy mitochondrial function [2]. The importance of oxidative metabolism is highlighted by the high mitochondrial content of heart muscle cells, where up to 40% of the cellular volume can consist of mitochondria.

The high energy requirements of heart manifest also in a high mtDNA content. The few studies including adult human cardiac muscle propose a mean copy number of 6970±920 to 9235±5457 mtDNA per cell, with individual numbers ranging from 4 000 to up to 34 000 [3], [4]. For comparison, skeletal muscle cells, depending on the muscle, have copy numbers ranging from 1000 to 4000 mtDNA molecules per cell.

The human 16,569 bp mitochondrial genome is typically seen in cells as monomeric, double-stranded, covalently closed circles. However, according to our recent findings, a major fraction of human heart mtDNA exist as dimers and is organized in highly complex, branched molecules, containing sometimes dozens of genome equivalents [5]. These structures contain three- and four-way DNA junctions, indicating active replication and recombination, somewhat resembling DNA replication in T-even phages [6], malarial mtDNA [7] as well as some plant and yeast mitochondria [8], [9]. Also the human brain has a subpopulation of molecules having similar features, while other tissues and cultured cells replicate their mtDNA using a standard theta-mechanism but incorporating extensive stretches of ribonucleotides on the lagging-strand [10], [11].

For unknown reasons, the recombining-replicating forms of mtDNA seem at present to be a feature specific to human heart and they have not been found in mice, rats, rabbits or pigs [5]. Despite the fact that neither the mitochondrial DNA binding protein TFAM nor Twinkle can promote recombination in cultured mammalian cells [12], [13], the overexpression of either TFAM or the mtDNA helicase Twinkle in transgenic mouse hearts induces an increase in complex DNA forms and recombination junctions, indicating that recombination mediating mechanisms occur also in other mammals under specific conditions [5]. Interestingly, the TFAM overexpressing mice are protected against the adverse effects of ischemia suggesting that mtDNA could play a role in cardiac remodeling [14].

To further characterize conditions that modify of the complex human heart mtDNA organization, we have analyzed the effect of age and the consequences of different cardiac diseases on heart mtDNA maintenance in human heart. While we did not observe any qualitative changes in the organization or replication of mtDNA in classified heart diseases, we discovered that cardiomyocytes of human newborns have a simple mtDNA organization, with relatively little recombination intermediates and dimeric molecules. The adult-type maintenance mode is acquired in the first years of life, correlating with a drastic increase in mtDNA copy number.

A dominant negative Twinkle mutation (aa 352–364 duplication), associated with a late-onset myopathy, completely abolished the adult-type mtDNA recombination phenotype and organization, including dimeric molecules without any effect on mtDNA copy number. In contrast, a mutation in the catalytic subumit of DNA polymerase γ (PolGα;p.-G848S/p-S1104C) mutation influenced only the maintenance of dimeric mitochondrial genomes in adult human heart. These results suggest that Twinkle is directly involved in the maintenance of mtDNA recombination in adult human heart.

## Results

In order to detect aging related changes in mtDNA replication intermediates (mtRIs) we analyzed 24 healthy left ventricular heart samples from individuals aged 0–83 years by using the two-dimensional agarose gel electrophoresis (2D-AGE) methodology (Table 1). We also compared the heart mtRI patterns from individuals with a fatal heart disease or with various mitochondrial disorders, to the mtRI patterns of age-matched healthy hearts (Table 2). We screened only for clear-cut effects, such changes in the replication mode or drastic reduction in the relative quantities of molecules with four-way junctions.

**Table 1:** Details of the left ventricular heart muscle samples from healthy controls used in this study.

	Age	Sex	Cause of death	Additional disease	Notes
**Controls**	1 day	M	Transposition of the great arteries (TGA)	No	
	1 day	F	Intrauterine hypoxia	No	
	1 day	M	Primary pulmonary hypertension	No	
	5 mo	M	Meningitis	No	
	2	M	Accidental poisoning	No	
	3	M	Gastroenteritis	No	
	8	F	Gastroenteritis	No	
	9	F	Grand mal status epilepticus	Congenital brain trauma	
	12	M	Traffic accident	No	
	12	F	Pneumonia	No	
	16	M	Opportunistic mycosis caused by medication	No	
	17	F	Traffic accident	No	
	18	M	Traffic accident	No	
	19	M	Suicidal poisoning	No	
	22	M	Suicide by rifle	No	
	23	M	Suicide by shotgun	No	
	31	F	Suicide by motor vehicle	No	
	40	M	Suicidal poisoning	No	
	52	M	Traffic accident	No	
	53	M	Suicide by handgun	No	
	55	M	Accidental suffocation	No	
	71	M	Drowning	No	
	78	F	Suicide by strangulation	No	
	83	F	Suicide by strangulation	No	

Age, sex, cause of death and disease status specified. M = Male, F = Female.

**Table 2:** Details of the left ventricular heart muscle samples of individuals with a fatal diagnosed heart disease or a mitochondrial disorder.

	Age	Sex	Cause of death	Additional disease	Notes	
**Myocardial infarction**	46	M	Acute infarct	Ischemic cardiomyopathy		
	50	F	Acute infarct	Ischemic cardiomyopathy		
	58	M	Old infarct	Coronary heart disease		
	64	M	Old infarct	Coronary heart disease		
	65	M	Old infarct	Coronary heart disease		
	73	F	Acute infarct	Ischemic cardiomyopathy		
	83	F	By-pass surgery complication	Coronary heart disease		
	84	M	Acute infarct	Coronary heart disease		
**Cardiomyopathies**	24	F	DCM	No		
	35	F	HCM	No	Ealier cardiac arrest, successful resuscitation	
	44	M	HCM	Coronary heart disease		
	48	M	HCM	No		
	55	M	Unspecified cardiomyopathy	No		
	59	M	Unspecified cardiomyopathy	No		
	76	M	DCM	No		
	81	M	Ischemic cardiomyopathy	Coronary heart disease		
	84	M	DCM, Mitral stenosis	No		
**Mitochondrial**	1 day	F	Lactic acidosis, cardiac failure	Fatal neonatal mitochondrial disease	homoplasmic m.1624C>T *MT-TV* mutation, [15]	
	34	F	Cardiac arrest following seizure	MELAS	m.3243A>G *MT-TL1* mutation, unpublished	
	41	F	Pneumonia	KSS	4.0 kb single, large-scale mtDNA deletion, unpublished	
	50	M	Cardiomyopathy	Hearing loss, myopathy, heart failure	m.7472Cins + m.7472A>C *MT-TS1* mutation, [35]	
	52	F	Pneumonia	MELAS	m.3243A>G MT-TL1 mutation, unpublished	
	55	M	myocardial infarction	Myopathy/ataxia	m.14709T>C *MT-TE* mutation, [36]	
	59	F	Pneumonia	MELAS	m.3243A>G MT-TL1 mutation, unpublished	
	59	M	Aspiration pneumonia	Parkinsonism, extrapyramidal syndrome	multiple mtDNA deletions - compound POLG mutations, [18]	
	60	M	Respiratory insufficiency	adPEO	Twinkle dup352–364 [16]	
	60	F	Respiratory insufficiency	adPEO	Twinkle dup352–364 [16]	
	73	F	Suicide	adPEO	Twinkle dup352–364 [16]	

DCM =  Dilating cardiomyopathy, HCM =  Hypertrophic cardiomyopathy.

### Lack of junctional mtDNA forms in newborn human heart

**Figure 1:**
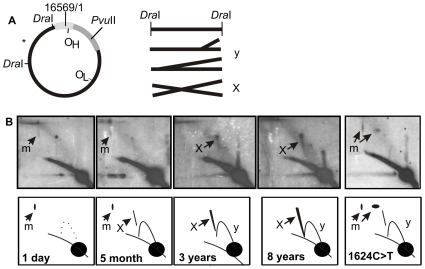
Specific change in human heart mtDNA replication and recombination in early childhood. (A) A diagram of human mtDNA showing the analyzed *Dra*I ND5 probed fragment (nt 12,271-16,010, probe location denoted with *) and resulting molecular forms of the fragment after digest. Simple, dsDNA replication fork migrate on the Y-arc (y), four-way junctional molecules on the X-arc (X). (B) 2D-AGE of the *Dra*I ND5 fragment from a one-day old newborn, five-month old, three-years old, eight-years old human heart mtDNA and a one-day old neonate with mitochondrial disease due to a homoplasmic m.1624C>T mutation. Notice the accumulation of X-arc signal and an overall increase in replication intermediates with aging. Degraded slow-moving Y-arc structures (m), indicators of ribonucleotide-incorporating replication, can be seen on some of the samples (see also [5].).

**Figure 2:**
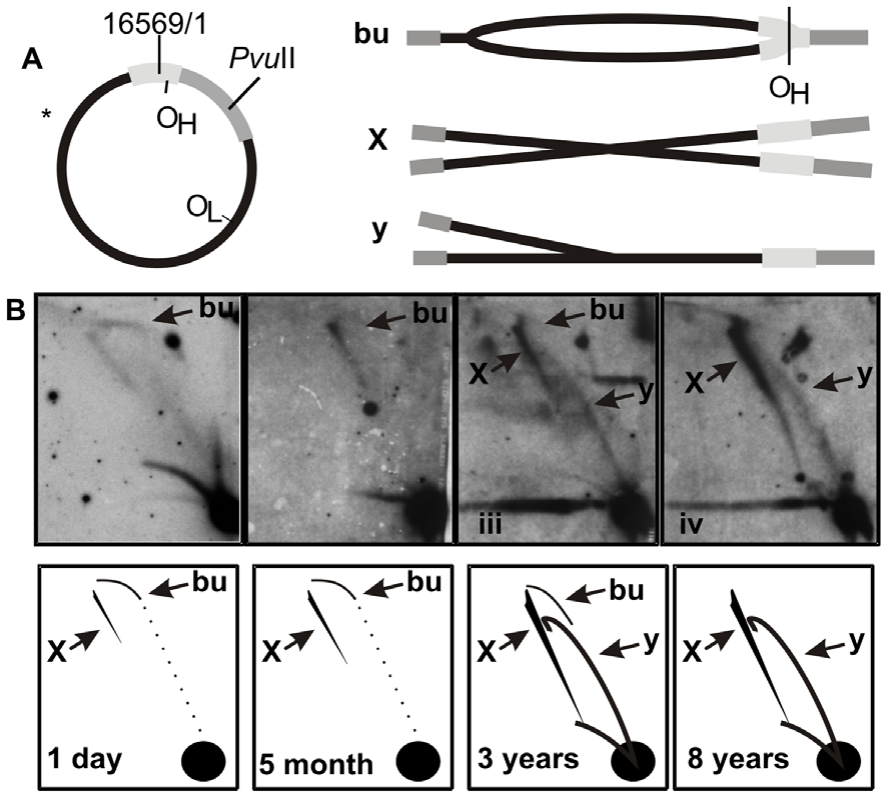
Theta-type mtRIs in infant heart. (A) A diagram of human mtDNA showing the probe location (*), the *Pvu*II cut site (nt 2565) and the resulting molecular forms after digest. In the case of unidirectional theta-replication originating from the O_H_, the molecules containing replication bubbles form almost complete bubble-arc (bu) on the 2D-AGE. Y- or Y-like arc likely results from initiation outside O_H_ but can in the case of human heart mtDNA also contain more diverse forms of mtRIs (see [5] for further explanation). (B) Single-cut *Pvu*II 2D-AGE analysis of the samples in Figure 1. Faint bubble-arcs seen at younger age are gradually replaced by X- and Y-forms of much stronger intensity. Heart mtRIs from children aged 8–10 years show no difference to the adult mtRI forms.

When analyzing the age dependency of junctional mtDNA forms in healthy humans, we noticed that notably less mtRIs existed in newborns and that four-way junctional mtDNA molecules – a feature typical to adult heart – were undetectable (Figure 1). Instead, some high-molecular weight forms, resembling a modified slow-moving Y-arc, could be seen (Figure 1B: m). These arcs were similar to the ones seen in tissues having ribonucleotide-incorporating replication intermediates [5]. The mtDNA replication pattern of a case with a fatal, neonatal mitochondrial disease with biventricular hypertrophic cardiomyopathy due to a pathogenic homoplasmic m.1624C>T *MT-TV* (see [15] for detailed description), was comparable with the age-matched controls (Figure 1B). Further analysis of mtRIs from young children of various ages showed a gradual shift to the adult-type mtDNA pattern (Figure 2). The four-way junctional forms seemed to increase already in the first years of life, being comparable with those of adults before the age of ten. Notably, unlike in adult human hearts, in 2D-AGE of *Pvu*II digested heart mtDNA of newborns, a faint bubble arc can be seen (Figure 2B: bu) indicating that at least some molecules are replicating via the theta-replication mode typical for cultured cells and most mammalian tissues [5]. It should be noted that also in normal mouse heart mtRIs patterns are weak and theta type replication bubbles can be best detected using single-cut 2D-AGE conditions [5].

### Twinkle mutation modifies the mtDNA replication mode in adult human heart

**Figure 3:**
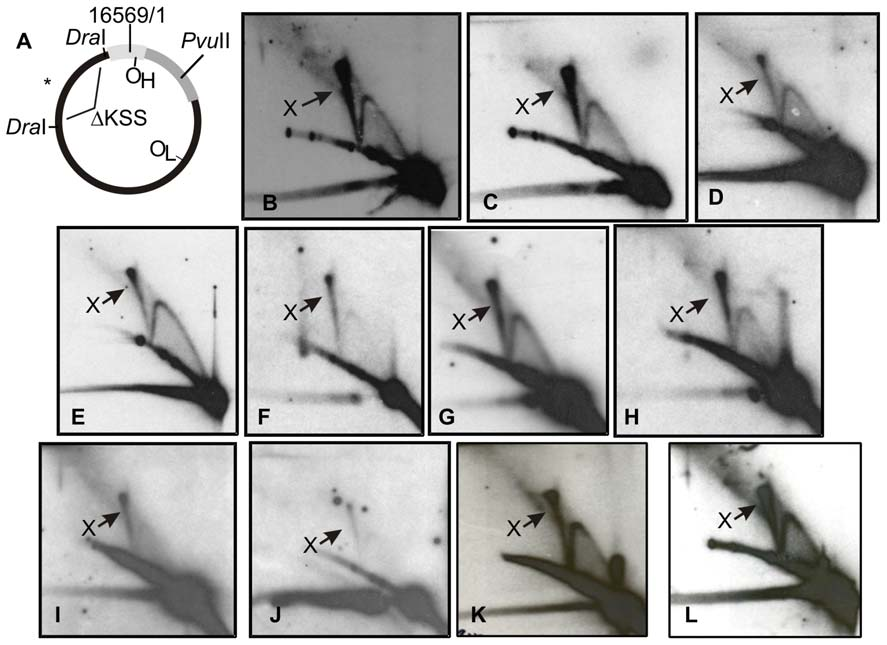
No qualitative change mtDNA replication and recombination intermediates in diseased human hearts. All analyzed samples retained the typical strong X-arc (X) and showed no evidence of ribonucleotide-incorporating replication intermediates. (A) A diagram of human mtDNA showing the analyzed *Dra*I ND5 probed fragment as earlier, followed by 2D-AGE panels of this representing (B) a male 46 years, acute ischemia, (C) male 84 years, chronic ischemia, (D) male 46, hypertrophic cardiomyopathy, (E) female 24, dilating cardiomyopathy, (F) mitochondrial cardiomyopathy, m.14709 T>C mutation, (G) mitochondrial cardiomyopathy (m.7472Cins + m.7472A>C), (H) m.3243A>G MELAS, (I) PolG (p.G848S/p. S1104C) mutation, (J) KSS (∼4.0 kb single mtDNA deletion, see also Figure S1), (K) a healthy control male, 19 years and (L) a healthy control female, 83 years.

**Figure 4:**
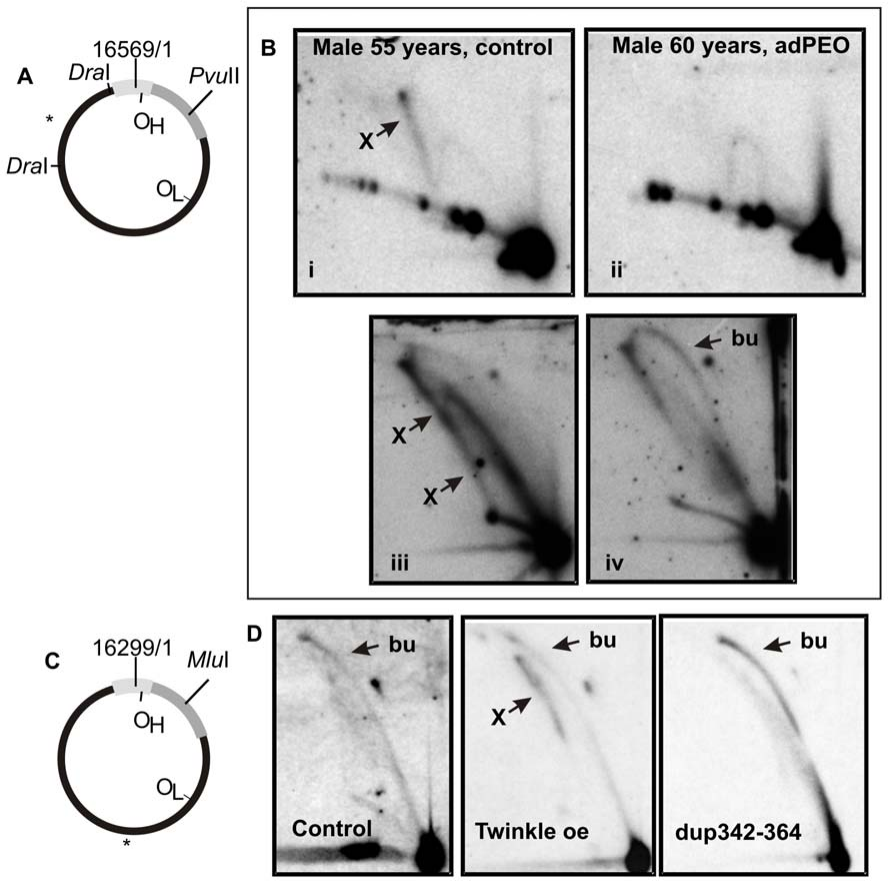
Theta-type mtDNA replication in heart muscle of adult adPEO patients with Twinkle dup352–364 mutation. (A) Human mtDNA showing the *Dra*I and *Pvu*II cut sites and probe locations. (B) Comparable exposures of 2D-AGE analysis of *Dra*I ND5 fragment (i–ii) and *Pvu*II (iii–iv) cut heart mtDNA from an age matched healthy control (i, iii) and adPEO patient (ii, iv). As a striking difference to the control samples, an almost complete absence of recombination intermediates (X) and a strong bubble arc (bu) in the adPEO heart muscle can be seen. (C) Mouse mtDNA showing *Mlu*I cut site (nt 1771) that is comparable to the human mtDNA *Pvu*II cut site location. (D) 2D-AGE of a *Mlu*I cut heart mtDNA from a control mouse, Twinkle overexpressor (oe) mouse and a mouse expressing the human Twinkle dup342-364 mutation. Whereas wild-type Twinkle enhances recombination junction formation, dup342-364 results in strong replication stalling phenotype in mouse heart. Unlike in mice, the overall intensity in the mtRIs of human adPEO patient heart is rather lower when compared to the age matched control hearts.

In contrast to the clear-cut differences observed in young children compared to the adult mtDNA, we were unable to detect any changes in the mtDNA replication or organization in diseased hearts, including cases with acute ischemic myocardial infarction without previous heart disease manifestation, old myocardial infarction with a long history of ischemic heart disease, as well as with cases with dilating and hypertrophic cardiomyopathy, ischemic cardiomyopathy and various mitochondrial disorders (Figure 3). Besides investigating patients with cardiac manifestations due to specific mtDNA point mutations, our series of mitochondrial disorders also included three patients with Twinkle dup352–364 mutation resulting in autosomal dominant progressive external ophtalmoplegia (adPEO) [16], [17], a patient with an extrapyramidal syndrome resulting from a compound heterozygous mutations affecting the α-subunit of the mtDNA polymerase, PolG (p.-G848S/p-S1104C, see [18]) and a patient carrying a 3978 bp single, large-scale mtDNA deletion (11657:15636), flanked by a 12 bp direct repeat, who presented with Kearns-Sayre syndrome (KSS). Of these patients, only the Twinkle dup352–364 cases showed a phenotype on 2D-AGE (Figure 4). In these samples almost complete abolishment of the recombination intermediates was seen (Figure 4B:ii), while the replication bubbles typical for theta-replication were retained (Figure 4B:iv). As mentioned earlier, unlike humans, mouse heart mtDNA does not have abundant recombination intermediates and mtRIs are seemingly similar as in other tissues [5] (Figure 4D). Whereas overexpression of wild-type Twinkle promote recombination of mouse heart mtDNA, mice carrying the Twinkle dup352–364 mutation show a strong mtDNA replication stalling phenotype, similar to the one observed in cultured cells expressing the same mutation [19]. The expression level of the transgene in these mice is comparable to – or even lower than would be expected in human heterozygous condition [20].

### Changes in heart mtDNA replication and organization correlate with copy number and with mutations affecting Twinkle or PolGα

**Figure 5:**
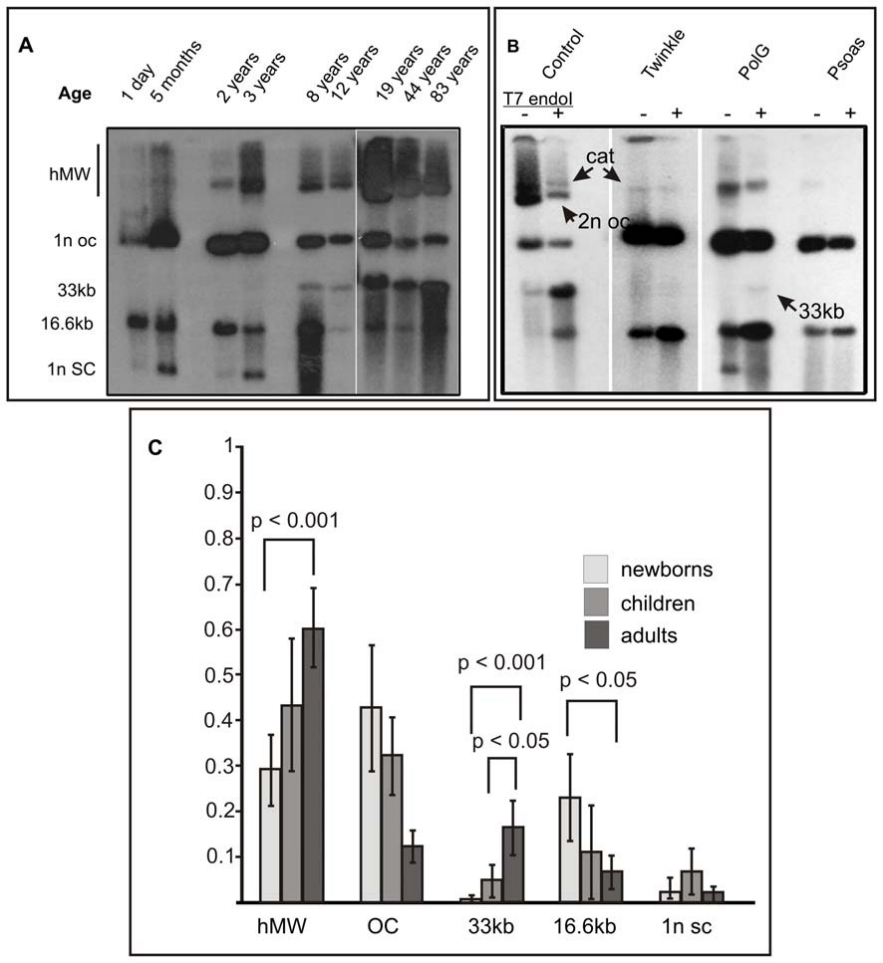
Human heart mtDNA topology changes in early childhood and in mitochondrial disorders involving mutations in Twinkle and PolG. The basis of the assignment of the different molecular forms is presented in [5]. (A) Newborns and young children have mainly monomeric open circular (1n oc), monomeric linear (16.6 kb) and monomeric supercoiled (1n sc) mtDNA molecules. Dimeric molecules, such as dimeric linears (33 kb) and complex mtDNA forms increase in early childhood. The quantities of different forms vary in adults and do not seem to depend on age or pathological status (data not shown). However, in an adult adPEO cases having Twinkle dup342-354 or with PolG (p.G848S/p. S1104C) compound mutation (PolG) the newborn-type mtDNA organization, including the monomeric supercoils are retained (B). T7 endonuclease I (T7 endoI) cleaves specifically branched DNA structures. Digestion of the heart DNA from the PolG patient shows that majority of the mtDNA molecules also in the branched structures are monomeric and there are around 14-fold less dimeric molecules than in the age matched control heart (male 44 years). In control heart, dimeric circular (2n oc) mtDNA molecules among the high molecular weight forms persist after T7 endoI, wheras they are not present in Twinkle adPEO patients and greatly decreased in the patient with mutant PolG. The other HMW forms being resistant to T7 endoI are catenanes (cat). As a comparison, in human skeletal muscle (ilio-psoas) there are very few T7 endo I sensitive molecules, showing that complex branched mtDNA forms are still present in the PolG mutant patient heart (See also Ref #5 for the assignment of the different molecular forms of human heart mtDNA). (C) Phosphoimager quantification of different mtDNA forms in cardiac muscle from one-day old newborns, young children (2–8 years) and adults. Statistically significant differences in high molecular weight (MW) forms, dimeric and monomeric molecules were detected (p-values; Students t-test). It should be noted that linear molecules are likely to arise due to artefactual breakage of mtDNA and can vary in *post mortem* samples depending on sample preservation.

When analyzing the topological organization of mtDNA, we noticed that the high molecular weight (HMW) forms and dimeric mtDNA molecules seen in adult human heart were undetectable in infants, but gradually increase together with the appearance of the recombination junctions (Figure 5A). In the adPEO patients with Twinkle mutation, almost all complex mtDNA forms were missing, including dimeric molecules (Figure 5B). The patient with PolG mutations seem to retain most junctional forms but has a drastic reduction in all dimeric mtDNA forms.

Overall, in the analyzed adult heart muscle samples there is some individual variation in the quantities of the different mtDNA forms, but this does not seem to be connected to the pathology or age. Curiously, in the case of the KSS patient with the ∼4.0 kb common deletion, deleted molecules cannot be detected without digesting the mtDNA with a restriction enzyme (Figure S1), indicating that the majority of the deletions in heart is probably associated with larger molecules.

**Figure 6:**
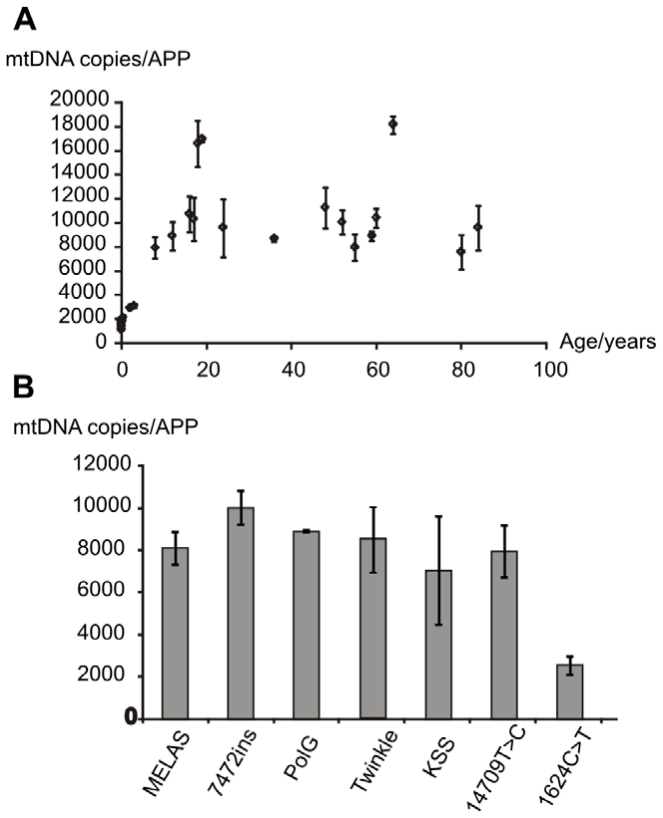
Increase in human heart mtDNA copy number during childhood correlates with the changes in molecular organization. (A) Five-fold increase in mtDNA copy number from newborns to teen-age. As with the mtDNA organization, individual variation can be seen in adults. Standard deviation represents three independent measurements of the same samples. (B) No significant difference in mtDNA copy number of PolG compound mutant patient heart, indicating that existence of dimeric mtDNA molecules is independent of copy number and quantity of branched mtDNA structures. With the exception of KSS, the mtDNA copy numbers were determined by qPCR against a single copy nuclear gene (APP). Because of the location of the *cytb* amplicon in the deleted region, copy number of the KSS was determined by Southern hybridization and absolute numbers were estimated by comparison with two control samples that were quantified also by qPCR (Figure S1, Text S1).

To see whether the increase in the complexity of mtDNA organization from newborns to adults also manifests as an increase in mtDNA copy number, a Real-Time quantitative PCR (qPCR) assay was performed (Figure 6). As expected, newborn babies seem to have almost five-fold lower heart mtDNA copy numbers than adults and the copy number steadily increased concomitantly with the changes in mtDNA organization and replication. Interestingly neither Twinkle nor the compound PolG mutations seemed to deplete mtDNA in heart. As Miller *et al.* [3], we did not observe any age dependency in human heart mtDNA copy number in adults.

## Discussion

### Relationship of heart mtDNA replication, organization and copy number

We report here that newborn human heart mtDNA has a relatively simple organization, lacking dimeric molecules and abundant recombination intermediates and bearing resemblance to the mtDNA of mouse heart or human skeletal muscle [5]. The adult type organization develops gradually in infancy with a concomitant rise in the mtDNA copy number. It is reasonable that these changes are related to the switching from fetal heart program to meet the metabolic requirements of adult heart.

As the highly complex branched forms of adult heart mtDNA harbor multiple copies of the genome it is likely that they also represent the nucleoid organization inside the mitochondria, where several copies may provide more chances for molecules to recombine. The idea of mtDNA density-induced recombination is further supported by the appearance of high molecular weight mtDNA forms and recombination intermediates in tissues with increased copy number in mice overexpressing Twinkle or TFAM. [5]. A similar phenomenon is seen in phage T4, where complex molecular networks are formed only when there are many genomes present in the same cell [6].

### The role of Twinkle and PolGα in heart mtDNA maintenance

We have earlier shown that the overexpression of wild-type Twinkle in mouse heart promotes recombination junction formation together with an increase in mtDNA copy number [5] (Figure 4D), whereas the Twinkle dup352–364 mutation has been shown to impair helicase activity, resulting in a strong replication stalling phenotype in cultured cells as well as in mice [19]. In cultured cells overexpression of the mutated helicase leads to rapid mtDNA copy number depletion, however in the heterozygous stage mice and humans typically do not show any mtDNA depletion, but accumulation of deleted mtDNA molecules in various tissues [16], [20]. The data presented here further suggests that the mutation does not have as severe deleterious effects on mtDNA replication as it has on recombination - resulting in almost complete abolishment of the otherwise abundant heart mtDNA recombination intermediates.

Mice carrying the Twinkle dup352–364 mutation did not show any cardiac phenotype [20]. However in some, but not all the Twinkle dup352–364 patients of the same family, asymptomatic, left cardioventricular hypertrophy, sinus bradycardia and ischemic changes were detected [16]. It is unclear if the heart phenotype of the patients is related to the Twinkle mutation. Obviously also the wild-type mice and adolescent humans can thrive without adult human-type mtDNA maintenance mode, meaning that the benefits of such a mode must be restricted to only very special physiological context.

Based on the data from various other genetic systems, one would expect replication stalling to result in an increase in double-strand breaks, which in turn are mostly repaired by recombination [21], [22]. If Twinkle's effect on recombination junction formation is as direct as the data suggest, the observed deleted mtDNA molecules in adPEO patients could equally be an end result of compromised recombination machinery instead of abortive replication [23]. A strong indication for such a repair system comes from the fact that the saturation of mitochondrial DSB repair machinery by a mitochondrially targeted endonuclease results in deleted mtDNA molecules that – unlike mtDNA deletions in healthy heart – do not have homology in the break-points, indicating that they are generated by non-homologous end joining [24].

In the adPEO patients the relationship between mtDNA copy number and structural maintenance is not as straight-forward as could be inferred from the data from the developmental changes in childhood. Twinkle seems to be required for the maintenance of the four-way junctions and complex mtDNA forms in adult heart, but it seems that this function is separate from the copy number control in this tissue. However, as we are only observing an end result in the adult patient hearts it is difficult to address whether some adaptive mechanisms have been involved.

Otherwise, the importance of Twinkle in mtDNA recombination is not surprising, as the maintenance of recombination intermediates requires a helicase [25], albeit generally different from replicative helicases. However, as many mitochondrial proteins have functional redundancy and no mammalian mitochondrial recombinases are known to-date it might well be that in certain cellular conditions Twinkle could carry out more functions than expected. It should be noted that via homology to T7 DNA-helicase Twinkle is included in the family of DnaB-helicase family and thus in turn related to the RecA family of recombinases [17], [26].

In the PolG (p. G848S/p.S1104C) mutant patient heart the mtDNA copy number is not dramatically affected in the heart of the patient and X-forms are present on the 2D-AGE panel (Figure 3I), however the dimeric linear 33 kb molecules as well as HMW mtDNA forms are almost completely absent (Figure 5). Most of the HMWs that can be resolved in regular agarose gel electrophoresis consist of dimeric circles [5] and the reduction in the HMW signal is mostly due to the absence of most dimeric forms. The p.G848S mutation is located in the catalytic domain of the protein and severely impairs the enzyme's polymerase activity *in vitro* [27]. As the Twinkle patients also showed complete absence of dimeric mtDNA molecules, this could indicate that they are primarily generated via recombination but later maintained by replication. As in mouse mitochondria the dimeric circular molecules take up to three times longer to replicate than monomers [28], the dimeric and larger genomes might simply be selected out when the mtDNA synthesis rate is reduced.

The p.G848S mutation in compound heterozygosity has been reported in patients with Progressive External Ophtalmoplegia (PEO) and Alpers-Huttenlocher syndrome with mtDNA depletion [29]–[31]. Similar to our case, the p.G848S mutation in compound with a heterozygous p.T251I mutation has been found in a family with autosomal recessive PEO and multiple mtDNA deletions in muscle [29]. It is not clear how the same mutation can be associated with either multiple deletions or depletion of mtDNA, but variability of clinical and molecular phenotypes seem to be common in PolG disease mutations [32].

The results from both the Twinkle and PolG patients provide some insight into the tissue specific features of many mitochondrial disorders. Overall speed of mtDNA replication synthesis, turnover, mtDNA organization and the existence of backup mechanisms, such as active recombination, could be limiting for many disease mutations of mtDNA maintenance proteins that do not show any phenotype in cultured cells or *in vitro* [19], [33]. These variables have been largely neglected when mitochondrial diseases have been studied. Simple Southern analysis of different topological forms of mtDNA could prove to be highly informative when characterizing the phenotype of mitochondrial disorders. More studies on the intrinsic and extrinsic factors affecting mtDNA replication and topology, especially in mouse models, will contribute to the understanding of the disease mechanisms and the healthy maintenance of mtDNA organization.

## Materials and Methods

### Autopsy series

The series comprises of left ventricular cardiac muscle samples from 24 autopsy cases (ages 0–83 years, Table 1) without any diagnosed heart disease, 17 cases with various heart diseases diagnosed at autopsy and 14 cases with a mitochondrial disease (Table 2). The samples originating from the Newcastle University were obtained with Institutional Ethical Approval and with a written consent of the family. The consent was given after the death and prior to the autopsy of the patient (within 4 hours of death). The samples from the University of Helsinki were collected with Institutional Ethical Approval and with verbal consent of next-to-kin. The consent was given after the death and prior to the autopsy of the patient (within 2–48 hours of death). The consent is documented in the medical records of the deceased. Verbal consent was the standard procedure at the time, acknowledged by the Institutional Ethical committee and following the national legislation. Most of the samples come from cases that have been previously published (see references in the Table 2). The rest of the myocardial samples were taken as part of the Tampere Coronary Study, approved by the Ethics Committee of Tampere University Hospital (DNO 1239/32/200/01) and the Finnish National Authority for Medico-legal Affairs.

### Transgenic animals

Twinkle overexpressor mice were as described in [5] and the mice expressing Twinkle dup352–364 as in [20].

### DNA extraction

Total DNA from frozen autopsy samples was extracted using proteinase K digestion followed by phenol chloroform extractions and ethanol precipitation [34].

### Enzymatic treatments and agarose gel electrophoresis

1D and 2D-AGE were performed as previously described [5], [17], [18]. Restriction digestions and other enzyme treatments were performed following manufacturers' recommendations [5]. For a better resolution of dimeric linear molecules on 1D-AGE, gels presented in figure 5 were 0.5% in contrast to 0.28–0.4% used in Pohjoismäki *et al.* [5].

### Southern blotting, radiolabelled probes and blot hybridization

Southern blotting and radioactive detection of DNA was performed as previously [5], [12], [34].

## Supporting Information

Text S1 Supplementary materials and methods.
(0.03 MB DOC)

Figure S1 MtDNA molecules having large scale deletion are not detected in topology gels in the case of KSS heart sample. (A) A diagram showing *Acc*I and *Eco*RI cut sites on human mtDNA. *Eco*RI cuts human mtDNA at locations 4121, 5274 and 12640. The 11657:15636 deletion diminishes *Eco*RI cut site at nt 12,640, giving rise to a 11.4 kb restriction fragment instead of a 8 kb one when probed with the OH probe. In order to estimate mtDNA copy number in the KSS case, the signal from the full length and deleted OH fragment was quantified against 18S nDNA signal using phophoimager. The result was matched against two age-matched controls whose mtDNA copy number was also measured by qPCR for absolute values (mtDNA copies per single-copy nuclear gene, APP). (B) The uncut 12 kb deletion cannot be detected with the same probe, even after disassembling the high-molecular weight mtDNA structures using topoisomerase IV (TIV) and T7 endonuclease I (T7). However, large amounts of heterogeneous molecules are released (seen as smear), suggesting that the deleted molecules are possibly associated with larger rearrangements. (C) 2D-AGE of the 4.8 kb *Acc*I fragment outside of the KSS deletion shows no difference to the age-matched control sample.
(0.26 MB PDF)
