# Supplementary material for: Developmental and Pathological Changes in the Human Cardiac Muscle Mitochondrial DNA Organization, Replication and Copy Number
Source: PLoS One. 2010 May 3;5(5):e10426. doi: 10.1371/journal.pone.0010426 (PMC2862702; doi:10.1371/journal.pone.0010426)
Supplement: Text S1 — Supplementary materials and methods. (0.03 MB DOC) [file pone.0010426.s001.doc]

**Text S1**

**Pohjoismäki *et al.* : Developmental and pathological changes in the human cardiac muscle mitochondrial DNA organization, replication and copy number**

**Supplementary materials and methods**

*Enzymatic treatments of mtDNA*

Topoisomerase IV (John Innes Enterprises) and T7 endonuclease I (New England Biolabs) treatments and gel conditions as described in Pohjoismäki *et al.* (1).

*Probes and hybridization*

OH, ND2 and 18S probes and Southern hybridization was performed as in Pohjoismäki et al. (1).

**References**

1. Pohjoismäki JL, Wanrooij S, Hyvärinen AK, Goffart S, Holt IJ, Spelbrink JN, Jacobs HT (2006) Alterations to the expression level of mitochondrial transcription factor A, TFAM, modify the mode of mitochondrial DNA replication in cultured human cells. Nucleic Acids Res. 34:5815-28.
