# Supplementary material for: Developmental and Pathological Changes in the Human Cardiac Muscle Mitochondrial DNA Organization, Replication and Copy Number
Source: PLoS One. 2010 May 3;5(5):e10426. doi: 10.1371/journal.pone.0010426 (PMC2862702; doi:10.1371/journal.pone.0010426)
Supplement: Figure S1 — MtDNA molecules having large scale deletion are not detected in topology gels in the case of KSS heart sample. (A) A diagram showing AccI and EcoRI cut sites on human mtDNA. EcoRI cuts human mtDNA at locations 4121, 5274 and 12640. The 11657:15636 deletion diminishes EcoRI cut site at nt 12,640, giving rise to a 11.4 kb restriction fragment instead of a 8 kb one when probed with the OH probe. In order to estimate mtDNA copy number in the KSS case, the signal from the full length and deleted OH fragment was quantified against 18S nDNA signal using phophoimager. The result was matched against two age-matched controls whose mtDNA copy number was also measured by qPCR for absolute values (mtDNA copies per single-copy nuclear gene, APP). (B) The uncut 12 kb deletion cannot be detected with the same probe, even after disassembling the high-molecular weight mtDNA structures using topoisomerase IV (TIV) and T7 endonuclease I (T7). However, large amounts of heterogeneous molecules are released (seen as smear), suggesting that the deleted molecules are possibly associated with larger rearrangements. (C) 2D-AGE of the 4.8 kb AccI fragment outside of the KSS deletion shows no difference to the age-matched control sample. (0.26 MB PDF) [file pone.0010426.s002.pdf]

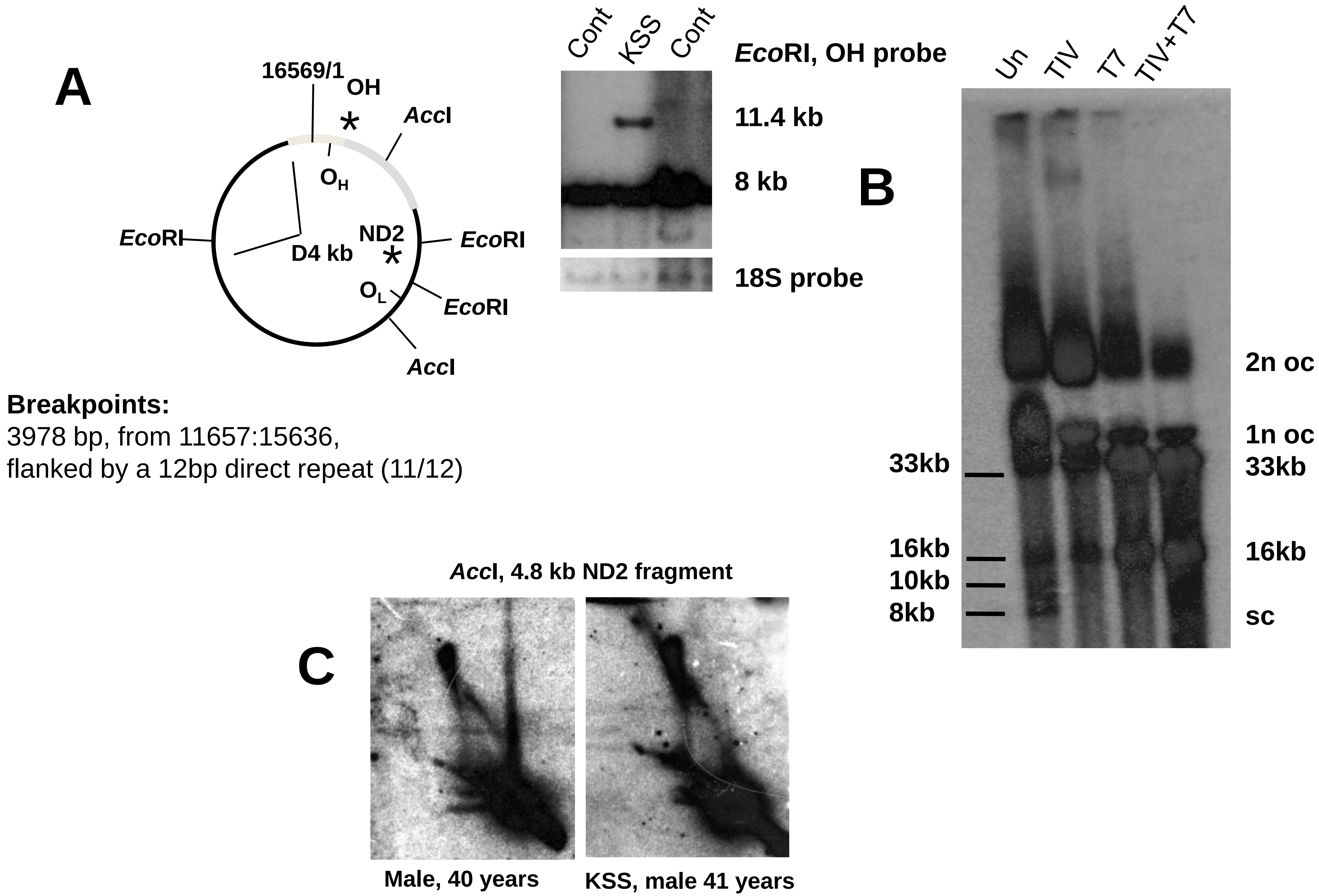
A
16569/1
OH
AccI
OH
EcoRI
EcoRI
ND2
D4 kb
OL
EcoRI
AccI
Breakpoints:
3978 bp, from 11657:15636,
flanked by a 12bp direct repeat (11/12)
Cont
KSS
Cont
EcoRI, OH probe
11.4 kb
8 kb
18S probe
B
Un
TIV
T7
TIV+T7
2n oc
1n oc
33kb
33kb
16kb
16kb
10kb
8kb
sc
AccI, 4.8 kb ND2 fragment
C
Male, 40 years
KSS, male 41 years

Figure S1
